# Prevalence, cessation, and geographical variation of smoking among middle-aged and elderly adults in China: A population-based study

**DOI:** 10.18332/tid/190247

**Published:** 2024-07-19

**Authors:** Qingjia Zeng, Chongyang Zhang, Feiyu Su, Yanli Wan, Wen-jun Tu, Hongpu Hu

**Affiliations:** 1 Institute of Medical Information/Medical Library, Chinese Academy of Medical Sciences and Peking Union Medical College, Beijing, China; 2 Department of Neurosurgery, Beijing Tiantan Hospital, Capital Medical University, Beijing, China

**Keywords:** smoking, smoking cessation, prevalence, associated factors, population-based

## Abstract

**INTRODUCTION:**

Smoking significantly burdens human health, contributing to an increasing incidence of mortality and morbidity. This study aims to explore the prevalence of smoking, cessation, and the association between various risk factors and smoking intensity measured in pack-years among Chinese adults.

**METHODS:**

During 2020–2021, the China Stroke High-risk Population Screening and Intervention Program (CSHPSIP) invited participants aged ≥40 years from 31 provinces in mainland China. This cross-sectional study presents the standardized prevalence of smoking and cessation across various demographics, including age, sex, residence, income, education level, BMI, and geographical region of residence. Multivariable logistic regression was used to examine the associations between smoking pack-years and related factors.

**RESULTS:**

Among 524741 participants (mean age: 61.9 ± 10.9 years; 41.1% male; 58.9% female), standardized smoking prevalence was 19.3% (95% CI: 19.2–19.4), with men (37.2%; 95% CI: 37.0–37.4) displaying significantly higher rates than women (1.3%; 95% CI: 1.2–1.3). Smoking cessation rate stood at 11.2% (95% CI: 11.0–11.4), with 11.3% (95% CI: 11.1–11.5) for men and 8.4% (95% CI: 7.5–9.2) for women. Urban residents and those with advanced education had lower smoking rates and higher cessation rates. Additionally, the dose-response relationship indicated a more pronounced association between higher smoking pack-years and elevated health risks, including hypertension (AOR=1.30; 95% CI: 1.24–1.36), diabetes (AOR=1.26; 95% CI: 1.20–1.33), hyperlipidemia (AOR=1.22; 95% CI: 1.16–1.28), heart disease (AOR=1.40; 95% CI: 1.26–1.54), and stroke (AOR=1.23; 95% CI: 1.10–1.36).

**CONCLUSIONS:**

This comprehensive study emphasizes the profound impact of smoking on health in Chinese adults, indicating the critical need for tailored cessation programs, particularly for middle-aged individuals, men, rural residents, and those with lower level of education.

## INTRODUCTION

Smoking represents a critical health hazard, significantly impacting human life and the global economy. Over the past three decades, smoking-related causes have resulted in >200 million deaths and an annual economic loss >1 trillion US dollars^1,2^. China alone has approximately 300 million smokers, accounting for nearly 40% of the world’s total tobacco consumption, and the number of deaths caused by smoking in China is the highest in the world^3^. Projections indicate up to 2 million tobacco-related deaths annually in the 2030s and around 3 million by 2050 in China, unless significant smoking cessation occurs^4^. These staggering statistics highlight the critical need for robust and effective anti-smoking strategies.

In 2017, smoking was identified as the primary cause of disability-adjusted life years (DALYs) in China^5^. A comprehensive prospective cohort study conducted in China revealed that smoking is associated with an elevated risk of 56 diseases, such as hypertension, diabetes, hyperlipidemia, heart disease, and stroke^6^. Previous studies have underscored the significant role of smoking as a risk factor for cardiovascular diseases^7^. Research shows that smoking increases the risk of pre-diabetes and diabetes in the general population^8^. Evidence suggests that current smokers exhibit a risk of stroke that is two to four times greater than that faced by lifelong non-smokers or individuals who ceased smoking more than a decade earlier^9^. Substantial evidence of moderate certainty affirms that smoking cessation is associated with a one-third risk reduction in recurrent cardiovascular ailments among individuals ceasing smoking post-diagnosis^10^.

Smoking as one of the most crucial modifiable health factors, and smoking cessation through intervention holds immense importance in enhancing the overall health status of the population. In 2011, the ‘Twelfth Five-Year Plan’ in China integrated the provisions for a complete ban on smoking in public areas^11^. By 2016, the ‘Healthy China 2030’ plan was launched, emphasizing a smoke-free environment. This ambitious plan set forth clear objectives: to enforce a complete ban on smoking in indoor public spaces and to reduce the smoking rate among those aged >15 years to a 20% by 2030^12^. On a local level, 107 Chinese cities have pioneered special smoking legislation for public places, often surpassing national benchmarks^13^. By August 2019, many mainland Chinese cities had adopted smoke-free laws, some fully compliant with WHO Framework Convention on Tobacco Control (WHO FCTC), with 9 of 21 cities banning smoking in all indoor workplaces, public places, and public transportation^14^.

However, current research on smoking and smoking cessation predominantly focuses on specific populations and regions, lacking comprehensive studies on smoking conditions among the entire population. To bridge this evidence gap, we examined data from the China Stroke High-risk Population Screening and Intervention Program (CSHPSIP) 2020–2021, a large-scale survey with national representation. This study aims to assess the prevalence of smoking and its cessation, and associations with sociodemographic factors among Chinese adults aged ≥40 years and encompassing specific subgroups.

## METHODS

### Study design and study participants

We conducted a nationwide cross-sectional study in 2021, collecting data from December 2020 to December 2021. Detailed information about the design, objectives, and survey methods of CSHPSIP is available in a previous publication^15^. The study’s methodology conformed to the STROBE (Strengthening the Reporting of Observational Studies in Epidemiology) guidelines^16^. The Bigdata Observatory Platform for Stroke of China (BOSC) is a comprehensive reporting system that records details of individuals aged ≥40 years from every hospital admission throughout China^17^. Covering 31 provinces, autonomous regions, and municipalities across China, this study invited community residents (those residing at the current location for six months or longer) registered at participating hospitals and screening sites to take part. The study protocol received approval from the Ethics Committee of Capital Medical University’s Xuanwu Hospital, in compliance with the Declaration of Helsinki (Reference No. 2012045). A detailed sampling design is described in Supplementary file Part 1.

**Figure 1:**
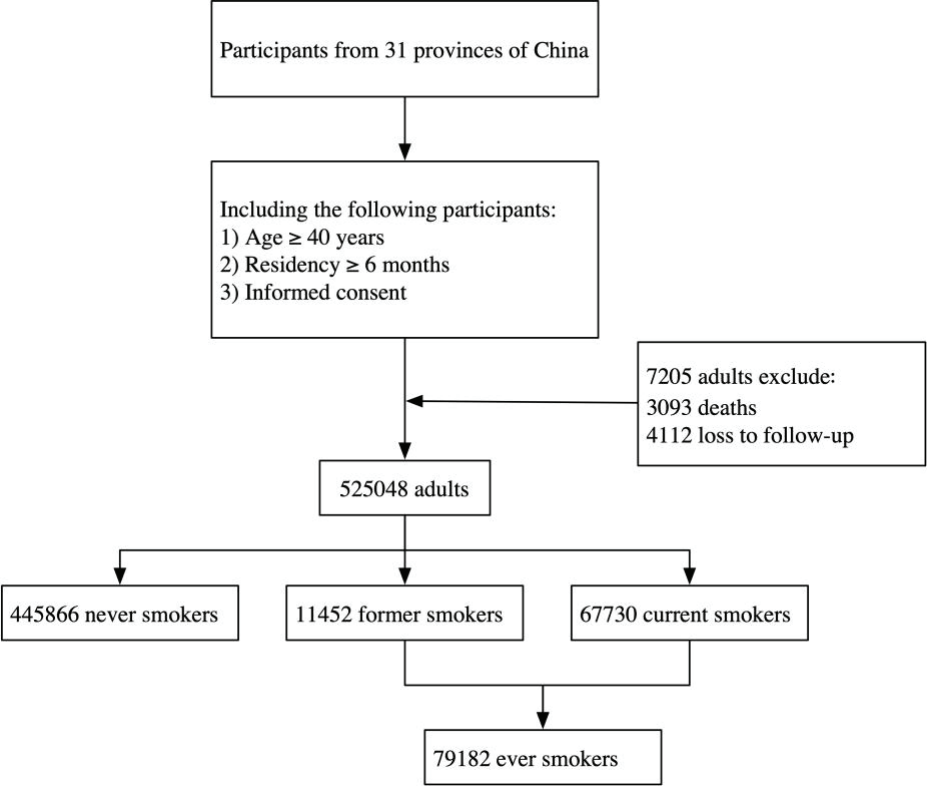
Study flowchart among Chinese Adults, 2021(N=525048)

After removing 4112 participants due to follow-up loss and 3093 due to deaths, the final number of individuals in our study was 525048 (Figure 1). Participants who had died were excluded because their deaths could confound the primary outcomes of interest related to smoking intensity and associated risk factors. The participants were divided into three groups: never smokers (N=445866), former smokers (N=11452), and current smokers (N=67730).

### Diagnostic criteria

Current smokers were identified as those who were smoking at the time of the survey, while former smokers were those who had quit smoking by the time of the survey. Both former and current smokers were collectively referred to as ever smokers. We determined the smoking prevalence by dividing the number of ever smokers by the total study population. The smoking cessation rate was calculated as the ratio of former to ever smokers. Additionally, current smokers provided details about the smoking duration, from the year they began smoking to the present, and the total number of cigarettes smoked daily. To quantify cumulative smoking exposure, we calculated pack-years, a metric commonly used in smoking-related research. Pack-years were calculated by dividing the daily number of cigarettes by twenty (average cigarettes per pack), then multiplying by the total years the person has smoked^18^.

### Covariates

Various sociodemographic factors were evaluated, including age, body mass index (BMI, kg/m^2^), education level, annual income level, and geographical region of residence. Age at the time of recruitment was treated as a continuous variable and additionally grouped into five age ranges: 40–49, 50–59, 60–69, 70–79, and ≥80 years. Hence, age was handled as a continuous and categorical variable. The education level was categorized as primary school or lower, middle school, high school, college or higher. Annual income was classified into four groups in RMB (1000 Chinese Renminbi about US$140): <5000, 5000–9999, 10000–19999, and ≥20000. Geographical regions were defined as follows: Northeast (Liaoning, Jilin, Heilongjiang), North (Beijing, Tianjin, Hebei, Shanxi, Inner Mongolia), Northwest (Shaanxi, Gansu, Qinghai, Ningxia, Xinjiang), Southwest (Chongqing, Sichuan, Guizhou, Yunnan, Tibet), South (Guangdong, Guangxi, Hainan), Central (Henan, Hubei, Hunan), and East (Shanghai, Jiangsu, Zhejiang, Anhui, Fujian, Jiangxi, Shandong). Additionally, the study examined a range of factors associated with health outcomes, including hypertension, diabetes mellitus, hyperlipidemia, obesity, transient ischemic attack (TIA), alcohol consumption, heart disease, stroke, and associated laboratory tests. Trained physicians diagnosed these conditions through clinical examinations, medical history assessments, and laboratory test results. All measurements of associated factors are described in Supplementary file Part 2.

### Statistical analysis

We assessed the characteristics of participants stratified by smoking status (never smokers, current smokers, and former smokers). Continuous variables were represented using means and standard deviations (SDs), and categorical variables were expressed in frequencies and percentages (%). The characteristics of the study population were compared using the Student’s t-test for continuous variables and the chi-squared test for categorical ones. The study applied sex- and age-standardized prevalence that was adjusted to align with the national demographic profile. This adjustment involved using various sampling weights, including those for the study’s design, the lack of response from some participants, and post-stratification (Supplementary file Part 3).

We applied multivariable logistic regression to calculate the odds ratios (ORs) and their 95% confidence intervals (95% CIs) for associations between smoking pack-years and various risk factors among current smokers, including hypertension, diabetes, hyperlipidemia, obesity, transient ischemic attack (TIA), heart disease, and stroke. Current smokers were stratified into three categories based on their pack-years: light smokers (1–20 pack-years), moderate smokers (21-40 pack-years), and heavy smokers (≥40 pack-years). To improve the accuracy of our findings, we adjusted for multiple potential confounders that could affect the relationships between smoking and these health conditions. Furthermore, we conducted subgroup analyses to explore the dose-response relationship between smoking pack-years and major health factors across different demographics, including age groups (middle-aged individuals, 40≤ and <60 years; and elders, aged ≥60 years), and genders (male, female).

Statistical analyses were conducted using SAS 9.4 and Python (version 3.9.12). All presented p-values were two-sided, with significance set at p<0.05.

## RESULTS

### Characteristics of participants

Among the 525048 participants aged ≥40 years, the average age was 61.9 years (SD=10.9). The vast majority (84.9%) were non-smokers, 12.9% were current smokers, and the remaining 2.2% were former smokers. Notably, the average age of former smokers was slightly higher at 66.0 years (SD=9.9). Furthermore, individuals who had never smoked or were former smokers were more likely to have a level of education of college or higher. Non-smokers generally had lower income levels compared to current and former smokers. Specifically, non-smokers had a higher proportion of individuals with an annual income of 0–5000 RMB compared to current and former smokers. In comparison, the proportion of non-smokers with an annual income of ≥20000 RMB was lower than that of current and former smokers. Almost all associated health factors were highest among former and, in some cases, among current smokers. Additionally, current and former smokers exhibited higher levels of systolic blood pressure (SBP), diastolic blood pressure (DBP), and homocysteine compared to non-smokers (Table 1).

**Table 1:** Demographic characteristics of study participants by different smoking status, among Chinese adults, 2021 (N=525048)

*Characteristics*	*All n (%)*	*Never smokers n (%)*	*Current smokers n (%)*	*Former smokers n (%)*	*p*
**Total**	525048 (100)	445866 (84.9)	67730 (12.9)	11452 (2.2)	<0.0001
**Age** (years), mean (SD)	61.9 (10.9)	61.9 (11.0)	61.3 (10.2)	66.0 (9.9)	<0.0001
**Age** (years)					
40–49	74188 (14.1)	63864 (14.3)	9617 (14.2)	707 (6.2)	<0.0001
50–59	164078 (31.2)	140242 (31.5)	21502 (31.7)	2334 (20.4)	
60–69	148866 (28.4)	124039 (27.8)	20887 (30.8)	3940 (34.4)	
70–79	107177 (20.4)	90475 (20.3)	13102 (19.3)	3600 (31.4)	
≥80	30739 (5.9)	27246 (6.1)	2622 (3.9)	871 (7.6)	
**BMI** (kg/m^2^)					
<18.5	11008 (2.1)	9012 (2.0)	1762 (2.6)	234 (2.0)	<0.0001
18.5–23.9	228636 (43.5)	195049 (43.7)	29321 (43.3)	4266 (37.3)	
24.0–27.9	211520 (40.3)	179115 (40.2)	27298 (40.3)	5107 (44.6)	
≥28.0	73884 (14.1)	62690 (14.1)	9349 (13.8)	1845 (16.1)	
**Education level**					
Primary school or lower	199700 (38.0)	171419 (38.4)	24106 (35.6)	4175 (36.5)	<0.0001
Junior high school	196745 (37.5)	165594 (37.1)	27048 (39.9)	4103 (35.8)	
High school	84480 (16.1)	71119 (16.0)	11324 (16.7)	2037 (17.8)	
College or higher	44123 (8.4)	37734 (8.5)	5252 (7.8)	1137 (9.9)	
**Annual income** (RMB)					
0–5000	129450 (24.7)	112620 (25.3)	14293 (21.1)	2537 (22.2)	<0.0001
5000–9999	80755 (15.4)	68804 (15.4)	10376 (15.3)	1575 (13.8)	
10000–19999	79884 (15.2)	67689 (15.2)	10469 (15.5)	1726 (15.1)	
≥20000	234959 (44.7)	196753 (44.1)	32592 (48.1)	5614 (49.0)	
**Geographical regions**					
North	84513 (16.1)	73405 (16.5)	9708 (14.3)	1400 (12.2)	<0.0001
Northeast	44398 (8.5)	37987 (8.5)	5833 (8.6)	578 (5.0)	
East	164623 (31.4)	140803 (31.6)	20398 (30.1)	3422 (29.9)	
Central	102415 (19.5)	85370 (19.1)	14477 (21.4)	2568 (22.4)	
South	29479 (5.6)	25331 (5.7)	3260 (4.8)	888 (7.8)	
Southwest	66361 (12.6)	54794 (12.3)	9474 (14.0)	2093 (18.3)	
Northwest	33259 (6.3)	28176 (6.3)	4580 (6.8)	503 (4.4)	
**Associated factors**					
Hypertension	248329 (47.3)	205661 (46.1)	35323 (52.2)	7345 (64.1)	<0.0001
Diabetes	114638 (21.8)	96953 (21.7)	14685 (21.7)	3000 (26.2)	<0.0001
Hyperlipidemia	212362 (40.4)	177669 (39.8)	29505 (43.6)	5188 (45.3)	<0.0001
Obesity	73884 (14.1)	62690 (14.1)	9349 (13.8)	1845 (16.1)	<0.0001
Transient ischemic attack (TIA)	8863 (1.7)	7573 (1.7)	1066 (1.6)	224 (2.0)	0.01
Alcohol consumption	81748 (15.6)	40353 (9.1)	36185 (53.4)	5210 (45.3)	<0.0001
Heart disease	26434 (5.0)	21911 (4.9)	3177 (4.7)	1346 (11.8)	<0.0001
Stroke	18533 (3.5)	14509 (3.3)	2975 (4.4)	1049 (9.2)	<0.0001
**Laboratory tests,** mean (SD)					
Systolic blood pressure (mmHg)	132.4 (17.2)	132.2 (17.1)	133.4 (17.4)	135.9 (17.7)	<0.0001
Diastolic blood pressure (mmHg)	80.4 (9.9)	80.1 (9.7)	82.2 (10.7)	82.5 (10.4)	<0.0001
Total cholesterol (mmol/L)	4.7 (1.2)	4.8 (1.2)	4.6 (1.1)	4.5 (1.1)	<0.0001
HDL cholesterol (mmol/L)	1.5 (0.6)	1.5 (0.6)	1.4 (0.5)	1.4 (0.4)	<0.0001
Fasting plasma glucose (mmol/L)	5.4 (1.7)	5.4 (1.7)	5.3 (1.7)	5.5 (1.7)	<0.0001
Homocysteine (μmol/L)	15.2 (8.4)	14.8 (8.1)	17.5 (10.0)	17.1 (8.9)	<0.0001
Glycated hemoglobin (HbA1c)	5.6 (1.2)	5.6 (1.2)	5.6 (1.2)	5.7 (1.3)	<0.0001

RMB: 1000 Chinese Renminbi about US$140.

### Prevalence of smoking and smoking cessation

**Figure 2:**
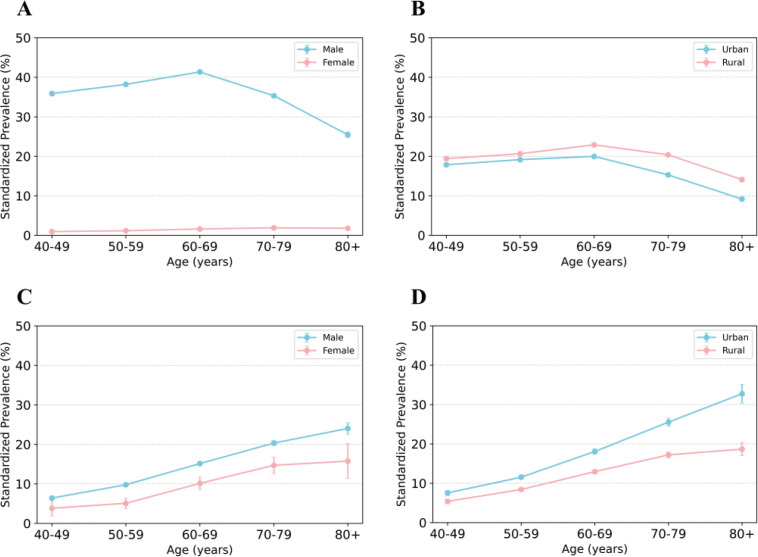
Standardized prevalence of smoking and smoking cessation for population aged 40 years and above (%); A and B: smoking rate by sex and residence; C and D: smoking cessation rate by sex and residence

As shown in Table 2, the standardized smoking prevalence among Chinese adults aged ≥40 years was 19.3% (95% CI: 19.2–19.4). Significant gender differences were observed: 37.2% (95% CI: 37.0–37.4) of men smoked compared to 1.3% (95% CI: 1.2–1.3) of women. The smoking cessation rate was 11.2% (95% CI: 11.0–11.4), with rates of 11.3% (95% CI: 11.1–11.5) for men and 8.4% (95% CI: 7.5–9.2) for women. Urban residents and those from South China exhibited commendable progress in tobacco control, reflected by lower smoking rates of 18.0% (95% CI: 17.8–18.1) and 16.7% (95% CI: 16.3–17.2), respectively, and elevated cessation rates of 13.2% (95% CI: 12.8–13.5) and 17.0% (95% CI: 15.9–18.2). The age-specific smoking prevalence displayed a bell curve, starting at 18.7% (95% CI: 18.4-19.0) for those aged 40–49 years, peaking at 21.7% (95% CI: 21.4–21.9) for 60–69 years, and dropping to 12.0% (95% CI: 11.6–12.3) for those aged ≥80 years. However, older individuals, regardless of sex or residence, were more likely to quit smoking, as evidenced by the rising trend in smoking cessation rates with age (Figure 2). Smoking prevalence increased with income, starting at 15.7% (95% CI: 15.5–15.9) for the 0–5000 RMB group and peaking at 21.5% (95% CI: 21.3–21.6) for those with income ≥20000 RMB.

**Table 2:** Standardized prevalence of smoking and smoking cessation in Chinese adult population, 2021 (N=525048)

*Characteristics*	*Standardized prevalence of smoking % (95% CI)*	*p*	*Standardized prevalence of smoking cessation % (95% CI)*	*p*
**Overall**	19.3 (19.2–19.4)		11.2 (11.0–11.4)	
**Age** (years)		<0.0001		<0.0001
40–49	18.7 (18.4–19.0)		6.4 (5.9–6.8)	
50–59	20.0 (19.8–20.2)		9.6 (9.3–10.0)	
60–69	21.7 (21.4–21.9)		15.0 (14.5–15.4)	
70–79	18.3 (18.1–18.6)		20.0 (19.4–20.6)	
≥80	12.0 (11.6–12.3)		23.3 (21.9–24.7)	
**Sex**		<0.0001		<0.0001
Male	37.2 (37.0–37.4)		11.3 (11.1–11.5)	
Female	1.3 (1.2–1.3)		8.4 (7.5–9.2)	
**Residence**		<0.0001		<0.0001
Urban	18.0 (17.8–18.1)		13.2 (12.8–13.5)	
Rural	20.4 (20.2–20.5)		9.8 (9.6–10.1)	
**Annual income** (RMB)		<0.0001		<0.0001
0–5000	15.7 (15.5–15.9)		13.1 (12.6–13.7)	
5000–9999	18.0 (17.7–18.2)		11.3 (10.7–11.8)	
10000–19999	19.7 (19.4–20.0)		11.2 (10.6–11.7)	
≥20000	21.5 (21.3–21.6)		10.5 (10.1–10.8)	
**Education level**		<0.0001		<0.0001
Primary school or lower	17.6 (17.4–17.7)		12.2 (11.8–12.6)	
Junior high school	20.2 (20.0–20.3)		10.1 (9.8–10.5)	
High school	21.5 (21.2–21.8)		11.1 (10.5–11.6)	
College or higher	18.6 (18.3–19.0)		12.7 (11.9–13.5)	
**BMI** (kg/m^2^)		<0.0001		<0.0001
<18.5	21.8 (21.0–22.5)		9.1 (7.9–10.4)	
18.5–23.9	17.9 (17.7–18.0)		9.6 (9.3–9.9)	
24.0–27.9	20.0 (19.8–20.2)		12.2 (11.8–12.5)	
≥28.0	21.4 (21.1–21.7)		12.8 (12.1–13.4)	
**Geographical regions**		<0.0001		<0.0001
North	17.2 (17.0–17.5)		7.9 (7.4–8.4)	
Northeast	18.2 (17.8–18.5)		8.1 (7.5–8.8)	
East	17.0 (16.8–17.2)		10.8 (10.4–11.2)	
Central	20.8 (20.6–21.1)		12.1 (11.6–12.5)	
South	16.7 (16.3–17.2)		17.0 (15.9–18.2)	
Southwest	25.2 (24.8–25.5)		15.2 (14.6–15.9)	
Northwest	20.4 (20.0–20.8)		5.0 (4.4–5.6)	

The standardized prevalence was calculated using sampling weights that were multiplied by design, non-response, and poststratification weights. Poststratification weights were adjusted for residence, geographical location, sex, and age using the 2010 China census data. RMB: 1000 Chinese Renminbi about US$140.

Conversely, increased income correlated with reduced cessation rates. Interestingly, both the highly educated (college or higher) and the least educated (primary school or lower) reported lower smoking rates and increased cessation rates compared to those with intermediate education levels (junior high and high school). Furthermore, both underweight and obese participants (BMI <18.5 and ≥28.0 kg/m^2^, respectively) had elevated smoking rates relative to those with a BMI of 18.5–27.9, while cessation rates rose with increasing BMI.

The prevalence of smoking and smoking cessation exhibited regional variations across China, as illustrated in Table 3. Provinces like Sichuan (27.77%, 18.78%) and Fujian (26.70%, 19.22%) reported high smoking and cessation rates. Conversely, Heilongjiang (13.57%, 6.28%), Hebei (14.27%, 9.13%), and Jilin (15.54%, 4.22%) showed low rates for both metrics. Guizhou (31.61%, 5.77%), Qinghai (22.97%, 2.53%), Hunan (22.84%, 8.47%), and Inner Mongolia (22.06%, 6.90%) presented high smoking rates but low cessation rates. In contrast, provinces like Beijing (11.13%, 24.31%) and Guangxi (13.94%, 18.65%) demonstrated low smoking and high cessation rates. Regarding gender-specific smoking rates across provinces, Fujian (59.84% for men, 0.53% for women), Sichuan (54.46%, 1.49%), Yunnan (50.46%, 0.53%), and Ningxia (48.19%, 0.06%) displayed high smoking prevalence among men but low among women. Conversely, Tianjin (21.73% for men, 4.83% for women) and Jilin (23.63%, 7.96%) exhibited low smoking rates in men but higher rates in women. Chongqing reported low smoking rates for both genders (17.32% for men and 0.30% for women).

**Table 3:** Geographical variation in the prevalence of smoking and cessation in the Chinese adult population, 2021 (N=525048)

*Province*	*Total participants*	*Overall smoking rate % (95% CI)*	*Smoking cessation rate % (95% CI)*	*Male smoking rate % (95% CI)*	*Female smoking rate % (95% CI)*
Beijing	4666	11.13 (10.23–12.04)	24.31 (20.54–28.07)	24.50 (22.40–26.61)	1.66 (1.63–1.68)
Tianjin	10416	13.71 (13.05–14.37)	15.92 (13.97–17.87)	21.73 (20.51–22.94)	4.83 (4.81–4.86)
Hebei	29384	14.27 (13.87–14.67)	9.13 (8.16–10.11)	26.31 (25.52–27.10)	0.65 (0.64–0.65)
Shanxi	32457	18.33 (17.91–18.76)	5.20 (4.53–5.87)	34.91 (34.12–35.71)	0.41 (0.40–0.41)
Inner Mongolia	7590	22.06 (21.12–22.99)	6.90 (5.67–8.13)	41.71 (39.91–43.51)	5.23 (5.22–5.25)
Liaoning	19580	22.08 (21.50–22.66)	9.58 (8.57–10.60)	40.68 (39.59–41.77)	4.38 (4.37–4.39)
Jilin	7758	15.54 (14.74–16.35)	4.22 (3.08–5.36)	23.63 (22.14–25.12)	7.96 (7.93–7.98)
Heilongjiang	17060	13.57 (13.06–14.09)	6.28 (5.21–7.34)	22.58 (21.61–23.55)	3.61 (3.60–3.62)
Shanghai	4852	16.11 (15.07–17.14)	8.20 (6.19–10.21)	31.17 (29.16–33.17)	0.85 (0.84–0.86)
Jiangsu	41484	19.18 (18.80–19.56)	9.92 (9.20–10.64)	38.92 (38.19–39.66)	0.53 (0.52–0.53)
Zhejiang	11470	19.74 (19.01–20.47)	11.01 (9.63–12.40)	39.51 (38.14–40.89)	0.38 (0.38–0.39)
Anhui	6710	14.22 (13.38–15.06)	12.53 (10.12–14.93)	29.97 (28.24–31.69)	0.28 (0.28–0.29)
Fujian	5441	26.70 (25.52–27.87)	19.22 (16.93–21.51)	59.84 (57.75–61.93)	0.53 (0.52–0.54)
Jiangxi	33147	20.29 (19.86–20.73)	10.93 (10.11–11.76)	39.47 (38.63–40.30)	1.38 (1.37–1.38)
Shandong	61519	13.22 (12.95–13.49)	10.26 (9.56–10.97)	25.39 (24.87–25.92)	0.50 (0.50–0.51)
Henan	37505	18.03 (17.64–18.42)	19.32 (18.22–20.42)	35.44 (34.68–36.20)	0.22 (0.22–0.23)
Hubei	13115	19.21 (18.53–19.88)	10.71 (9.37–12.05)	38.69 (37.37–40.01)	1.37 (1.35–1.38)
Hunan	51795	22.84 (22.48–23.20)	8.47 (7.93–9.02)	43.03 (42.38–43.67)	1.08 (1.08–1.09)
Guangdong	11793	20.94 (20.20–21.67)	17.24 (15.65–18.83)	43.22 (41.79–44.65)	0.61 (0.60–0.62)
Guangxi	11668	13.94 (13.31–14.56)	18.65 (16.63–20.67)	29.43 (28.11–30.76)	0.15 (0.14–0.15)
Hainan	6018	12.61 (11.77–13.45)	12.59 (9.77–15.40)	28.78 (26.88–30.68)	0.41 (0.40–0.42)
Chongqing	14485	8.52 (8.06–8.97)	13.06 (11.05–15.08)	17.32 (16.34–18.30)	0.30 (0.30–0.31)
Sichuan	31438	27.77 (27.28–28.27)	18.78 (17.82–19.75)	54.46 (53.58–55.33)	1.49 (1.48–1.49)
Guizhou	4713	31.61 (30.28–32.94)	5.77 (4.45–7.10)	54.11 (51.96–56.26)	3.11 (3.09–3.14)
Yunnan	15418	26.50 (25.80–27.19)	10.99 (9.87–12.12)	50.46 (49.25–51.67)	0.53 (0.52–0.53)
Tibet	307	12.38 (18.98–20.19)	12.59 (12.28–12.90)	18.38 (36.84–39.15)	2.75 (2.66–2.84)
Shaanxi	16503	19.58 (18.98–20.19)	2.14 (1.58–2.70)	37.99 (35.64–38.68)	0.39 (0.38–0.39)
Gansu	8791	20.72 (19.87–21.57)	5.80 (4.59–7.01)	37.16 (35.64–38.68)	0.82 (0.81–0.83)
Qinghai	2380	22.97 (21.27–24.66)	2.53 (0.88–4.17)	39.64 (36.59–42.70)	0.69 (0.67–0.70)
Ningxia	4319	21.38 (20.15–22.60)	13.87 (10.94–16.80)	48.19 (45.83–50.55)	0.06 (0.06–0.07)
Xinjiang	1266	17.96 (15.84–20.07)	20.61 (14.97–26.25)	23.82 (20.68–26.96)	3.06 (2.95–3.17)

### Relationship between pack-years and risk factors

Using multivariable logistic regression, we analyzed the associations between pack-years and various risk factors, as detailed in Table 4. Participants who smoked for 21–40 pack-years exhibited an increased likelihood of hypertension (AOR=1.08; 95% CI: 1.04–1.11), diabetes (AOR=1.08; 95% CI: 1.03–1.12), and hyperlipidemia (AOR=1.11; 95% CI: 1.07–1.15) compared to those who smoked for 1–20 pack-years (reference). For those who smoked more than 40 pack-years, the risk was significantly higher for several conditions: hypertension (AOR=1.30; 95% CI: 1.24–1.36), diabetes (AOR=1.26; 95% CI: 1.20–1.33), hyperlipidemia (AOR=1.22; 95% CI: 1.16–1.28), heart disease (AOR=1.40; 95% CI: 1.26–1.54), and stroke (AOR=1.23; 95% CI: 1.10–1.36), compared to those who smoked for 1–20 pack-years.

**Table 4:** Association between risk factors and pack-years* for current smokers in the Chinese adult population, 2021(N=67730)

*Characteristics*	*21–40 pack-years (N=23049)*		*>40 pack-years (N=10032)*	
	*AOR (95% CI)*	*p*	*AOR (95% CI)*	*p*
Hypertension	1.08 (1.04–1.11)	0.00	1.30 (1.24–1.36)	<0.0001
Diabetes	1.08 (1.03–1.12)	0.04	1.26 (1.20–1.33)	<0.0001
Hyperlipidemia	1.11 (1.07–1.15)	0.00	1.22 (1.16–1.28)	<0.0001
Obesity	0.97 (0.92–1.02)	0.05	1.05 (0.98–1.13)	0.06
TIA	0.90 (0.78–1.03)	0.10	1.01 (0.85–1.21)	0.44
Heart disease	1.04 (0.96–1.13)	0.05	1.40 (1.26–1.54)	<0.0001
Stroke	1.05 (0.96–1.14)	0.16	1.23 (1.10–1.36)	0.00

* Reference category: 1–20 pack-years (N=34649).

AOR: adjusted odds ratio. AORs (95% CIs) were calculated using a multivariate logistic regression model, adjusted for age, gender, residence, income level, education level, and geographical region of residence. The significance of each predictor was assessed using the Wald chi-squared test, and p-values were derived accordingly. TIA: transient ischemic attack.

Subgroup analysis, stratified by gender and age, examined the relationship between risk factors and pack-years, as detailed in Table 5. For males, the dose-response relationship was consistent with the overall population trends. Females faced increased heart disease and stroke risks with higher pack-years, most notably for those with >40 pack-years. Notably, females with greater than 40 pack-years also faced increased risks for hypertension and diabetes, a trend not observed in the 21–40 pack-year group compared to the reference category of 1–20 pack-years. In the cohort of younger participants (<60 years), higher pack-years was correlated with a considerable rise in the risk of hypertension, diabetes, hyperlipidemia, and heart disease.

**Table 5:** Subgroup analysis of risk factors and pack-years* for current smokers in the Chinese adult population, 2021 (N=67730)

*Characteristics*	*Males (N=64037)*				*Females (N=3693)*			
	*21–40 pack-years (N=22299)*		*>40 pack-years (N=9811)*		*21–40 pack-years (N=750)*		*>40 pack-years (N=221)*	
	*AOR (95% CI)*	*p*	*AOR (95% CI)*	*p*	*AOR (95% CI)*	*p*	*AOR (95% CI)*	*p*
Hypertension	1.08 (1.05–1.12)	0.00	1.30 (1.24–1.36)	<0.0001	0.94 (0.80–1.11)	0.10	1.50 (1.13–2.01)	0.00
Diabetes	1.07 (1.03–1.12)	0.04	1.26 (1.19–1.33)	<0.0001	1.11 (0.91–1.35)	0.42	1.48 (1.08–2.01)	0.03
Hyperlipidemia	1.11 (1.07–1.15)	0.00	1.23 (1.17–1.29)	<0.0001	1.17 (0.99–1.38)	0.31	1.11 (0.83–1.46)	0.87
Obesity	0.98 (0.93–1.03)	0.06	1.05 (0.98–1.13)	0.07	0.90 (0.71–1.15)	0.18	1.21 (0.81–1.75)	0.23
TIA	0.90 (0.79–1.04)	0.10	1.03 (0.86–1.23)	0.35	0.70 (0.32–1.40)	0.99	0.49 (0.08–1.64)	0.47
Heart disease	1.00 (0.92–1.09)	0.20	1.37 (1.23–1.52)	<0.0001	1.59 (1.23–2.05)	0.02	1.80 (1.19–2.66)	0.01
Stroke	1.01 (0.93–1.10)	0.07	1.20 (1.08–1.33)	0.00	1.64 (1.19–2.26)	0.02	1.81 (1.07–2.91)	0.01
***Characteristics***	***Participants aged <60 years (N=31118)***				***Participants aged ≥60 years (N=36612)***			
	***21–40 pack-years (N=10613)***		***>40 pack-years (N=2184)***		***21–40 pack-years (N=12436)***		***>40 pack-years (N=7848)***	
	***AOR (95% CI)***	***p***	***AOR (95% CI)***	***p***	***AOR (95% CI)***	***p***	***AOR (95% CI)***	***p***
Hypertension	1.11 (1.05–1.16)	0.01	1.35 (1.23–1.47)	<0.0001	0.98 (0.94–1.03)	0.17	1.03 (0.98–1.09)	0.14
Diabetes	1.16 (1.09–1.23)	0.02	1.40 (1.26–1.56)	<0.0001	0.97 (0.92–1.03)	0.11	1.08 (1.02–1.15)	0.00
Hyperlipidemia	1.13 (1.07–1.18)	0.01	1.37 (1.25–1.50)	<0.0001	1.07 (1.02–1.13)	0.01	1.13 (1.07–1.19)	<0.0001
Obesity	1.00 (0.94–1.07)	0.22	1.20 (1.07–1.34)	0.00	0.88 (0.82–0.95)	0.01	0.94 (0.86–1.03)	0.98
TIA	0.84 (0.68–1.03)	0.07	1.11 (0.77–1.56)	0.28	0.95 (0.79–1.14)	0.53	1.01 (0.82–1.24)	0.74
Heart disease	1.34 (1.14–1.57)	0.04	1.57 (1.18–2.05)	0.02	0.87 (0.79–0.96)	0.04	1.00 (0.89–1.11)	0.22
Stroke	1.19 (1.00–1.41)	0.47	1.62 (1.23–2.10)	0.00	0.92 (0.84–1.01)	0.61	0.89 (0.79–1.00)	0.17

* Reference category: 1–20 pack-years: Males (N=31927), Females (N=2722), <60 years (N=128321), ≥60 years (N=16328).

AOR: adjusted odds ratio. AORs (95% CIs) were calculated using a multivariate logistic regression model. The age-specific model was adjusted for gender, residence, income level, education level, and geographical region of residence. The gender-specific model was adjusted for age, residence, income level, education level, and geographical region of residence. The significance of each predictor was assessed using the Wald chi-squared test, and p-values were derived accordingly. TIA: transient ischemic attack.

Conversely, for those aged ≥60 years, the risk increase was more modest, with only hyperlipidemia showing a marked rise in the two higher pack-year groups and diabetes in the highest pack-year group. Obesity risk was greater in participants aged <60 years who smoked more than 40 pack-years but lower in those aged >60 years who smoked 21–40 pack-years, compared to the 1–20 pack-year reference group. Moreover, the risk of transient ischemic attack (TIA) did not show a significant correlation with the intensity of smoking in any demographic subgroup.

## DISCUSSION

This research is among the most comprehensive studies assessing the prevalence, determinants, and cessation of smoking in a nationally representative sample of Chinese adults aged ≥40 years. The 2018 China Adult Tobacco Survey documented an overall smoking prevalence of 26.6%, distributed as 50.5% for males, 2.1% for females, 28.9% in rural areas, and 25.1% in urban. However, our study reported a diminished overall prevalence of 19.3%, including 37.2% in men, 1.3% in women, 20.4% in rural regions, and 18.0% in urban settings. Additional Chinese research has noted higher smoking rates compared to our results^19,20^. Comparable smoking prevalence rates were recorded in other Asian countries, such as Thailand (20.7%) and South Korea (20.8%)^21,22^. However, in comparison to developed nations like the United Kingdom (12.9%) and the United States (11.5%), the smoking rate we observed was notably higher^23,24^. Such discrepancies might arise from variations in tobacco control programs and legislative measures in these nations. The study indicates that smoking rates in China are significantly higher among men compared to women, a disparity much larger than the gender gap observed in Europe^25^. This difference reflects the cultural acceptance in East Asia, where smoking is more socially acceptable for men than for women^26^. In our study, the smoking cessation rate was 11.2%. Compared to other regions, we noted that the smoking cessation rate in our study was slightly lower than in the United Kingdom^27^ (14.0%) and Japan^28^ (15.3%). Various factors motivate smokers to quit. Policies and legislation that enforce the prohibition of smoking in public places play a significant role in encouraging cessation. Further, the accessibility of diverse cessation aids, including counseling services, nicotine replacement therapies, and support groups, offers invaluable assistance in the quitting journey^29^.

To examine the relationship between social demographics and both smoking habits and cessation, factors such as education level, culture, and economic development may be influential^30^. Urban residents and individuals with higher level of education tend to smoke less and have higher cessation rates, consistent with the research of Meza et al.^31^. This trend is likely driven by greater health awareness and health consciousness among educated populations. Moreover, urban areas generally benefit from better infrastructure and healthcare facilities and are often the focus of health awareness campaigns, including smoking prohibition initiatives. Participants in South China had a lower smoking rate and a higher cessation rate, likely due to their higher socio-economic status, which leads to better health awareness, education, and access to smoking cessation resources^32^. Regions like Beijing demonstrate the impact of stringent tobacco control policies. The lower prevalence of smoking in these areas underscores the effectiveness of robust public health policies, comprehensive awareness campaigns, and strategic public health investments.

Additionally, the data indicate an increased likelihood of smoking cessation with advancing age, consistent with the result of Najafipour et al.^33^. When smokers recognize the adverse health effects of smoking, they may become more motivated to quit. This presents an ideal opportunity for doctors and family members to encourage smoking cessation, particularly in patients suffering from smoking-related diseases.

Our results also indicate that smoking more than 40 pack-years substantially increases the risk of developing serious health conditions such as hypertension, diabetes, hyperlipidemia, heart disease, and stroke. The adverse effects of chronic tobacco smoking on blood pressure can be attributed to nicotine and carbon monoxide, two primary compounds found in tobacco. Nicotine causes both vasoconstriction and vasoparalytic effects, while chronic exposure to carbon monoxide can lead to irreversible changes in blood vessels^18^. Moreover, a Korean study demonstrated a dose-dependent effect of smoking on diabetes risk that persists even after cessation in those with over 14 pack-years of exposure, suggesting that the detrimental impacts of smoking on glucose metabolism are long-lasting and possibly irreversible^34^. Shah et al.^35^ also found that extensive smoking history significantly elevates blood lipid levels, aligning with our findings. These physiological changes, aggregated over years of smoking, heighten the risk of disease, rendering long-term smokers particularly susceptible to severe health outcomes.

### Strengths and limitations

Our study has several strengths, including a large and nationally representative sample size, well-validated questionnaires, and a rigorous quality control process. We collected a comprehensive range of data variables, encompassing smoking status, pack-years, demographic characteristics, and health conditions. This allowed for a deep understanding of the relationship between smoking and health outcomes. To strengthen the reliability of our results, we controlled for a variety of potential confounders, such as age, sex, BMI, and income. Our findings highlight a dose-response correlation between smoking severity and conditions such as hypertension, diabetes, hyperlipidemia, heart disease, and stroke. These insights emphasize the severe health repercussions of smoking habits and can guide the formulation of more effective preventative and control measures. Moreover, the risks associated with smoking are not uniform across gender or age groups, highlighting the need for targeted public health interventions and smoking cessation programs.

However, certain limitations should be considered when interpreting our findings. First, due to our study’s cross-sectional design, there exists a potential for reverse causality; thus, our data reveals associations, not necessarily causative links. Longitudinal studies would be needed to address this concern. Second, the reliance on self-reported smoking status, without biochemical validation, might introduce biases such as recall and social desirability, potentially affecting the accuracy of smoking reports. Third, while we have accounted for various factors, we may have overlooked crucial determinants like age at smoking initiation, attempting and motivation to quit, and specific tobacco product use, all of which might have health implications.

## CONCLUSIONS

This study represents the most comprehensive nationwide analysis of smoking prevalence and cessation behaviors to date in China. Its significance lies in its potential to inform public health initiatives and policy-making in China. By identifying specific subgroups with high smoking rates and low cessation, this research offers a strategic framework for tailored prevention and cessation initiatives. Additionally, this study deepens our understanding of the association between various risk factors and smoking intensity measured in pack-years. These insights significantly enrich our understanding of smoking dynamics in China, paving the way for targeted, data-driven methods to reduce smoking prevalence and improve health outcomes across diverse population groups.

## Supplementary Material



## Data Availability

The data supporting this research are available from the authors on reasonable request.
