# Supplementary material for: Prevalence, cessation, and geographical variation of smoking among middle-aged and elderly adults in China: A population-based study
Source: Tob Induc Dis. 2024 Jul 19;22:10.18332/tid/190247. doi: 10.18332/tid/190247 (PMC11259031; doi:10.18332/tid/190247)
Supplement: Supplementary file 1 [file TID-22-133-s1.pdf]

Supplementarys

Appendix 1. Sampling design

In this study, a two-stage stratified cluster sampling was employed to ensure representativeness of the national population of mainland China. Initially, cities were categorized into three types: developed, developing, and undeveloped. This classification was based on several criteria, including per capita GDP, commercial resources, the city's role as a commercial center, the energy of its inhabitants, lifestyle variety, and potential for growth. Following this, a proportion of cities was selected from each category: 16 developed, 75 developing, and 89 undeveloped cities, to act as the primary sampling units.

The second stage shifted focus to the smaller units within these cities, targeting communities or villages with a population exceeding 2,000 residents aged 40 and above. From each city, one such community or village was chosen randomly. The inclusion criteria for participants were age 40 and above, a residence duration of at least six months in the area, and voluntary consent to partake in the study. Data collection was conducted through face-to-face interviews using a standardized questionnaire at the designated hospital facilities. Demographic data (such as age, gender, and type of residence [either urban or rural]) for participants in each province and city were expected to align with the information in the Sixth National Population Census of China.[1] Any community or village with a response rate below 85% was not included in the study to ensure the data's reliability. Additionally, any screening location with fewer than 400 participants or an unbalanced gender ratio (above 1.4 or below 0.5) was excluded to maintain the study's validity.

---

[1] National Bureau of Statistics of China. Tabulation on the 2010 Population Census of the People’s Republic of China[J]. China Statistical Press, 2012.

Appendix 2. Measurements of variables

Trained interviewers from the stroke base hospital or community hospital visited each household to collect informed consent forms and arrange face-to-face interviews and physical examinations. During the structured interviews, demographic details were obtained from each subject using a standardized questionnaire to ensure the accuracy of the data collection.

The variables were meticulously defined to facilitate their precise identification. Hypertension was categorized as having a systolic blood pressure of 140 mm Hg or higher, or a diastolic blood pressure of 90 mm Hg or above. A fasting plasma glucose level of 7.0 mmol/L or greater was the criterion for a diabetes mellitus diagnosis. Hyperlipidemia was determined through specific fasting plasma markers, including total cholesterol levels of 6.22 mmol/L or higher, triglyceride levels of 2.26 mmol/L or more, and high-density lipoprotein cholesterol levels below 1.04 mmol/L. Obesity was identified with a body mass index (BMI) of 28 kg/m^2 or greater, adhering to the standards for Chinese adults. The study also considered transient ischemic attack (TIA) and heart disease by reviewing medical histories. Futhermore, stroke was diagnosed with certification or imaging evidence (CT/MRI) from a secondary or higher-level medical institution, specifically those at Level II or above.

Participants were also invited to attend physical measurements. Procedures for recording weight, height, and blood pressure adhered to a universally accepted protocol. For laboratory tests, fasting serum samples from participants were analyzed to measure indicators such as glucose, homocysteine, cholesterol, high-density lipoprotein, and glycated hemoglobin (HbA1c). These analyses were performed using the HP-AFS/3 automatic immunoassay system A3 Specific Protein Analyzer. Test results were directly uploaded to the Bigdata Observatory platform for Stroke of China (BOSC).

Appendix 3. Sample weights

We developed sample weights to account for multistage sampling design and post-stratification. The sample weights were determined through the following steps:

1. Base weights for multi-stage design (Wdesign)

Stratifications: East/ Central /West * Urban/Rural = 6 stratum

A1=Number of districts/counties in different stratum in the sample

B1=Number of districts/counties -level administrative districts in each stratum

Wdesign=B1/A1, assign the weight Wdesign of each stratum to the corresponding case of each stratum

2. Post-stratification weights (Wps)

Stratifications: gender (2 levels) * geographic region (6 levels: North China, Northeast China, East China, Central South, Southwest, Northwest) *age group (9 levels: 40-44, 45-49, 50-54, 55-59, 60-64, 65-69, 70-74, 75-79, 80+) =108 stratum

A2=The sum of the case weights Wdesign of each stratum of the sample

B2=Number of people by stratum in the 2010 Sixth census

Wps=B2/A2, assign the weight Wps of each stratum to the corresponding case of each stratum

3. Final weights (Fw)

Fw= Wdesign* Wps, assign the weight (Fw) to the corresponding case

Post-stratification adjustments considered several factors like whether participants lived in urban or rural areas, their location within specific geographical regions of China (such as the northeast, north, northwest, southwest, south, central, or east), their gender, and their age group, categorized into five brackets ranging from 40 to over 80 years old. These categories were informed by data from the Sixth National Census of China. The purpose of implementing these weighting methods was to make sure the study's results would accurately reflect the broader population of China, thus enhancing the reliability and applicability of the findings by addressing potential biases that might have arisen from how the study was designed or from participants not responding.

© **2024 Zeng Q. et al.**
